# Anemonin Attenuates RANKL-Induced Osteoclastogenesis and Ameliorates LPS-Induced Inflammatory Bone Loss in Mice *via* Modulation of NFATc1

**DOI:** 10.3389/fphar.2019.01696

**Published:** 2020-02-10

**Authors:** Huanhuan Hou, Qisheng Peng, Shaoming Wang, Yuxin Zhang, Jinjin Cao, Yuming Deng, Yingjian Wang, Wan-chun Sun, Hong-bing Wang

**Affiliations:** ^1^ Key Laboratory of Zoonosis, Ministry of Education, Department of Gynaecology and Obstetrics, China–Japan Union Hospital, Jilin University, Changchun, China; ^2^ Department of Endocrinology, Changchun People’s Hospital, Changchun, China; ^3^ Putuo District People’s Hospital, School of Life Sciences and Technology, Tongji University, Shanghai, China

**Keywords:** osteoclasts, anemonin, NFATc1, Blimp1, NF-κB, ERK1/2

## Abstract

Osteoporosis is a metabolic bone disease characterized by insufficient osteoblastic function and/or excessive osteoclastic activity. One promising strategy for treating osteoporosis is inhibiting excessive osteoclast resorbing activity. Previous studies have revealed that anemonin (ANE), isolated from various types of Chinese natural herbs, has anti-inflammatory and anti-oxidative properties. However, whether ANE regulates osteoclastogenesis is unknown. This study aimed to investigate the potential effect of ANE on osteoclastogenesis and inflammatory bone loss in mice. In *in vitro* studies, ANE suppressed RANKL-stimulated osteoclast differentiation and function by downregulating the expression of osteoclast master transcriptor NFATc1, as well as its upstream transcriptor c-Fos, by decreasing NF-κB and ERK1/2 signaling. Interestingly, ANE did not change the phosphorylation and degradation of IκB-α and activation of JNK and p38 MAPKs. However, ANE repressed the phosphorylation of MSK-1 which is the downstream target of ERK1/2 and p38 MAPK and can phosphorylate NF-κB p65 subunit. These results implicated that ANE might suppress NF-κB activity *via* modulation of ERK1/2 mediated NF-κB phosphorylation. In addition, ANE directly suppressed NFATc1 transcription by inhibiting Blimp-1 expression, and the subsequent enhancement of the expression of NFATc1 negative regulators, Bcl-6 and IRF-8. Moreover, *in vivo* studies were conducted using an LPS-induced inflammatory bone loss mice model. Micro-CT and histology analysis showed that ANE treatment significantly improved trabecular bone parameters and bone destruction. These data indicate that ANE can attenuate RANKL-induced osteoclastogenesis and ameliorate LPS-induced inflammatory bone loss in mice through modulation of NFATc1 *via* ERK1/2-mediated NF-κB phosphorylation and Blimp1 signal pathways. ANE may provide new treatment options for osteoclast-related diseases.

## Introduction

Osteoporosis is a metabolic bone disease characterized by insufficient osteoblastic function and/or excessive osteoclastic activity during bone remodeling (Bono and Einhorn, 2003), which often lead to bone lysis diseases such as osteoporosis and periodontitis (Manolagas and Jilka, 1995). Accordingly, inhibiting the bone-forming process and/or bone-resorbing function is one of the most common and effective strategies for developing novel osteoporosis medications.

Osteoclasts are large multinucleated cells originating from hematopoietic myeloid precursors that undergo a series of differentiations and exhibit bone-resorbing activity through degradation of the bone matrix (Teitelbaum, 2000; Bar-Shavit, 2007). Macrophage Colony Stimulating Factor (M-CSF) and Receptor Activator of Nuclear Factor-κ B Ligand (RANKL) play an essential role in osteoclastogenesis; Upon binding to RANK, RANKL can recruit the adaptor molecule, TNF receptor associated factor 6, which correspondingly stimulates intracellular signaling pathways involving nuclear factor-κB (NF-κB) and mitogen activated protein kinases (MAPKS), eventually leading to the activation of NFATc1, and further prompts osteoclast-related gene expression (Khosla, 2001; Boyle et al., 2003; Kim et al., 2008; Nakashima and Takayanagi, 2011). B-cell lymphoma 6 (Bcl-6) and interferon regulatory factor-8 (IRF-8) are negative regulators of NFATc1 activity (Kim et al., 2007; Kiyomiya et al., 2015). These regulators are downregulated by B lymphocyte-induced maturation protein1 (Blimp1) (Nishikawa et al., 2010; Shin et al., 2014).

Natural compounds and their derivatives play a pivotal role in the development of new methods for osteoporosis treatment (Thummuri et al., 2018). Anemonin (ANE) is a pentacyclic triterpenoid mainly isolated from *Pulsatilla chinensis* (Bunge) Regel*, Clematis chinensis* Osbeck or *Drymaria diandra* Blume (Huang et al., 2008). Previous studies have reported that ANE possesses multiple pharmacological and natural activities such as anti-inflammatory and anti-allergy activities (Duan et al., 2006; Wang et al., 2017). ANE could suppress osteoarthritis progression *via* inhibition of NF-κB signaling (Wang et al., 2017). As NF-κB signaling is crucial, this study investigated whether ANE can prevent RANKL-induced osteoclastogenesis and evaluated the potential therapeutic properties of ANE in LPS-induced bone loss mice model.

## Materials and Methods

### Reagents

**Figure 1:**
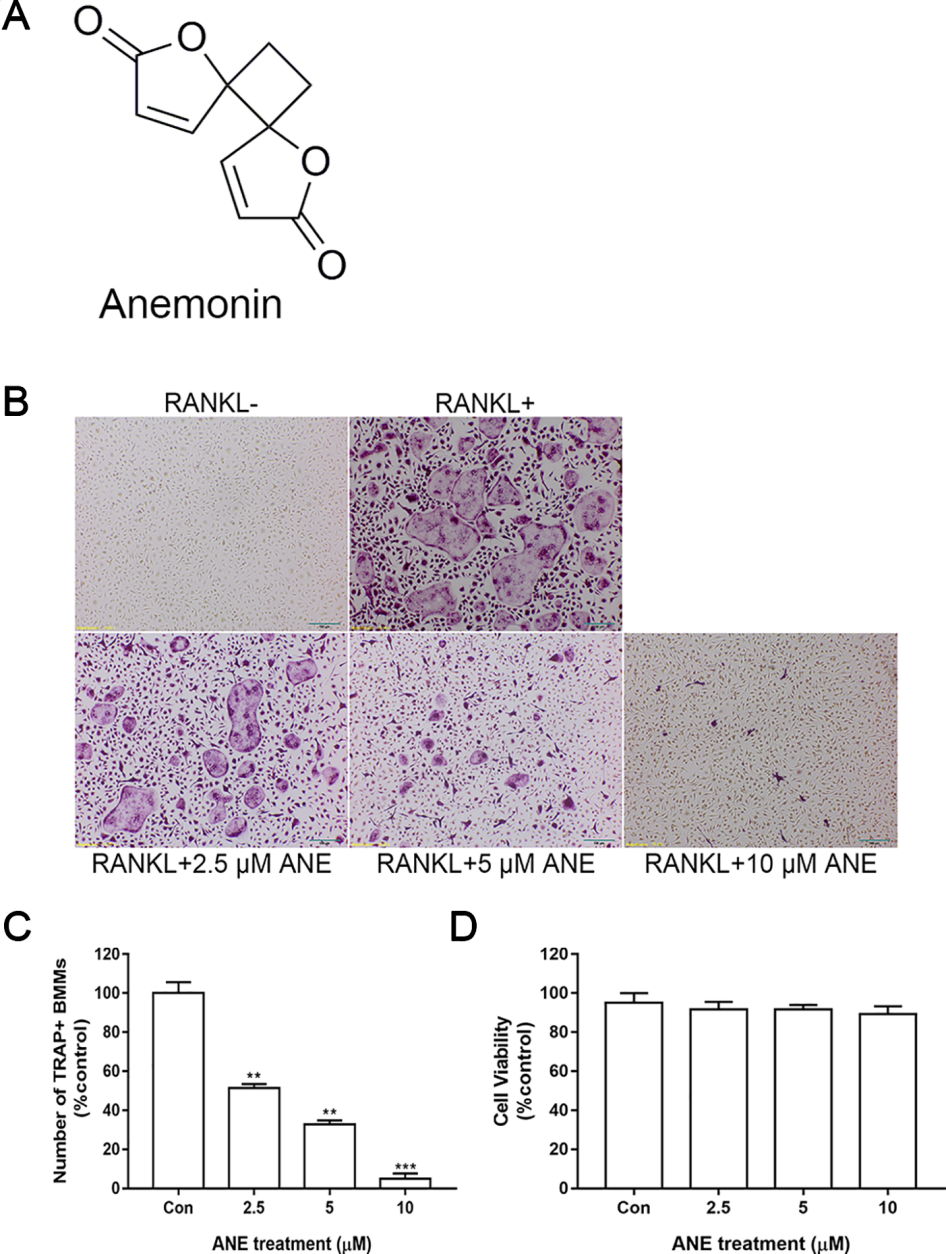
ANE Inhibits RANKL-Induced Osteoclast Formation. **(A)** Chemical structure of ANE. **(B)** BMMs (6 × 10^4^ cells/well) were treated with or without RANKL (100 ng/ml) and ANE (0-10 μM) in a 48-well plate for 4 days in the presence of M-CSF (40 ng/ml). Cells were fixed and stained *via* TRAP staining kit. **(C)** TRAP-positive multinucleated cells (≥ 3 nuclei) osteoclasts were counted as osteoclasts. RANKL-positive control group was set as 100%. **(D)** Cell viability was evaluated by CCK-8 assay. Scale bar = 100 μm. Data are presented as mean ± SD (n = 3). Statistical values were calculated using a Student’s t-test. **p < 0.01, and ***p < 0.001 vs. RANKL-positive control group. ANE, anemonin; BMMs, bone marrow-derived macrophages; M-CSF, Macrophage Colony Stimulating Factor.

Primary antibodies against p65, JNK, phospho-JNK, NFATc1, Phalloidin Cruz Fluor™ 594 conjugate, and c-Fos were obtained from Santa Cruz (CA, USA). Primary antibodies against phospho-p65 were obtained from Abcam (Cambridge, UK). Primary antibodies against phospho-p38, p38, and IĸB-α were purchased from ImmunoWay (Plano, TX, USA). Other antibodies were acquired from Cell Signaling Technology (Danvers, MA, USA). The RNeasy Mini Kit was obtained from QIAGEN (Frankfurt, German). The TRAP staining kit was purchased from Sigma-Aldrich (St. Louis, MO, USA). Fetal bovine serum (FBS) and α-MEM were obtained from Gibco (Rockford, IL, USA). ANE (purity > 98%; **Figure 1A**) was purchased from Shanghai Pureone Biotechnology (Shanghai, China), and dissolved in dimethyl sulfoxide (DMSO) followed by dilution with α-MEM medium. RANKL and M-CSF were procured from PeproTech (Rocky Hill, NJ, USA).

### Cell Culture and TRAP Staining Assay

Bone marrow cells were isolated from the femora and tibiae of 6-week-old male C57BL/6J mice, as previously described (Xu et al., 2016; Zhang et al., 2018). Extracted cells were cultured in α-MEM medium supplemented with 10% FBS, 100 U/ml penicillin and 100 μg/ml streptomycin in the presence of 40 ng/ml M-CSF at 37°C with 5% CO_2_. Non-adherent cells were collected the next day and incubated with α-MEM complete medium containing 40 ng/ml M-CSF for 2 days. Then, the cells were collected as bone marrow-derived macrophages (BMMs).

BMMs (6 × 10^4^ cells/well) were treated with or without 100 ng/ml RANKL and ANE (0–10 μM) in a 48-well plate for 4 days in the presence of M-CSF (40 ng/ml). α-MEM medium contains 0.1% DMSO. Then, cells were fixed and stained to detect TRAP activity as the TRAP staining kit’s protocol. TRAP-positive multinucleated cells (≥3 nuclei) osteoclasts were counted as osteoclasts.

### Cytotoxicity Assay

Briefly, BMMs (10^4^ cells/well) were added to 96-well plates in the absence or presence of 40 ng/ml M-CSF, 100 ng/ml RANKL and ANE (0-10 μM) for 48 h at 37°C in 5% CO_2_. The CCK-8 assay (Beyotime Institute of Biotechnology, Shanghai, China) was performed according to the manufacturer’s instructions and read at 450 nm using a microplate reader (Tecan, San Jose, CA, USA).

### Bone Pit Formation Assay

BMMs (2 × 10^5^ cells/well) were added to 24-well Osteo Assay Surface Plates (Corning, Tewksbury, MA, USA) and treated with or without RANKL (100 ng/ml) and ANE (0-10 µM) in the presence of M-CSF (40 ng/ml) for 4 days. Next, the cells were removed using 5% (w/v) sodium hypochlorite for 10 min and then stained using the modified von Kossa method (Xu et al., 2016; Zhang et al., 2018). 5% (w/v) silver nitrate was incubated in the wells avoiding light for 1 h at room temperature. And then 5% (w/v) sodium carbonate prepared in 10% formaldehyde was added to the wells, treated for 5 min at room temperature. Resorption pit area per well was imaged using a light microscope and counted by ImageJ software.

### Fibrous Actin (F-Actin) Ring Staining Assay

BMMs (6 × 10^4^ cells/well) were added to a 48-well plate and cultured with or without RANKL (100 ng/ml) and ANE (0-10 μM) in the presence of M-CSF (40 ng/ml) for 4 days. Cells were fixed with 4% paraformaldehyde for 30 min and permeabilized with 0.1% Triton X-100 in PBS for 10 min. Next, the cells were blocked with 5% BSA in PBS for 1 h and incubated with phalloidin for 1 h at 37°C. After washing with PBS, the cells were stained with DAPI and imaged using an Olympus BX35 fluorescent microscope.

### Real-Time PCR

BMMs (4 × 10^5^ cells/well) were added to 12-well plates in complete α-MEM medium, and cultured with or without RANKL (100 ng/ml) and ANE (10 μM) in the presence of M-CSF (40 ng/ml) for 4 days. Total RNA was extracted from cultured cells using the RNeasy Mini Kit and cDNA was synthesized using the Transcriptor First Strand cDNA Synthesis Kit (Roche, Basel, German). Real-Time PCR was performed using an ABI 7500 Fast Real-Time PCR System (Foster City, CA, USA). The primers used for the tested genes are detailed in **Table 1**; GAPDH was employed as a reference gene.

**Table 1:** Primer sequences for Real-Time PCR.

Gene name	Forward sequence 5’-3’	Reverse sequence 5’-3’
GAPDH	TCAAGAAGGTGGTGAAGCAG	AGTGGGAGTTGCTGTTGAAGT
NFATc1	GGAGAGTCCGAGAATCGAGAT	TTGCAGCTAGGAAGTACGTCT
c-Fos	CGGGTTTCAACGCCGACTA	TTGGCACTAGAGACGGACAGA
αv	TTGATTCAACAGGCAATCGAGA	AGCATACTCAACGGTCTTTGTG
DC-STAMP	TCCTCCATGAACAAACAGTTCCAA	AGACGTGGTTTAGGAATGCAGCTC
Atp6v0d2	CAGAGCTGTACTTCAATGTGGAC	AGGTCTCACACTGCACTAGGT
Blimp1	TTCTTGTGTGGTATTGTCGGG	TTGGGGACACTCTTTGGGTAGAGTT
IRF-8	CGGGGCTGATCTGGGAAAAT	CACAGCGTAACCTCGTCTTC
Bcl-6	ATGAGATTGCCCTGCATTTC	TTCTTCCAGTTGCAGGCTTT

### Western Blotting Assay

Cultured cells were lysed in RIPA buffer with 1% protease and phosphatase cocktail inhibitor (Sigma-Aldrich). Proteins (30–50 μg) were quantified using a bicinchoninic acid (BCA) assay kit, separated by 10% SDS-PAGE, and transferred to PVDF membranes. The membranes were blocked in 5% BSA for 2 h and then incubated with the indicated primary antibodies for approximately 12 h at 4°C. Next, the membranes were incubated with the corresponding secondary antibody. The membranes were imaged using the MicroChemi Chemiluminescence system (DNR, Jerusalem, Israel). Densitometry analysis of protein bands was performed using ImageJ software.

### LPS-Induced Inflammatory Bone Loss

Next, this study evaluated the therapeutic potential of ANE for inflammatory bone lysis disease *in vivo*. This project fully considered and protected the rights and interests of the study objects and was based on the principle of the Institutional Animal Ethics Committee of Jilin University. Healthy 6-week-old male C57BL/6J mice were separated into the following four groups (10 mice per group): sham group (saline), LPS group (only LPS and saline injected), low dose ANE group (LPS injected and 2 mg/kg ANE), high dose ANE group (LPS injected and 10 mg/kg ANE). All mice were subcutaneously intraperitoneally injected with saline and/or ANE was administered at one day prior to LPS (10 mg/kg) treatment, repeated every other day and continued for 10 days. The animals were then euthanized and the tibiae, femora, and blood were collected for further analysis. The fixed left femur sections were decalcified in 12% EDTA and embedded in paraffin for H&E and TRAP staining to examine bone erosion and osteoclast activity. The right femora were scanned through a high-resolution microcomputed tomography 35 µCT scanner (Scanco Medical AG, Bassersdorf, Switzerland) at an isotropic voxel size of 10 μm. The region of interest (ROI) is 1 mm from the growth plate. The bone parameters analyzed using Scanco Medical software included bone volume per tissue volume (BV/TV), trabecular separation (Tb.Sp), trabecular thickness (Tb.Th), and trabecular number (Tb.N.).

### Statistical Analysis

Data were expressed as mean ± SD of at least triplicate independent experiments. Statistical analyses were compared using a Student’s t-test or one way ANOVA using GraphPad Prism 7.0 (GraphPad Software, La Jolla, CA, USA). Results with p < 0.05 were considered statistically significant.

## Results

### ANE Inhibits RANKL-Induced Osteoclast Formation

First, the effect of ANE on osteoclast formation was assessed. ANE treatment decreased the number and size of TRAP-positive osteoclasts in a dose-dependent manner (**Figure 1B, C**). Next, to exclude the possibility cytotoxic effect of ANE on RANKL-induced osteoclasts, the viability of cells treated with different concentrations of ANE (0–10 μM) for 48 h was measured using the CCK-8 assay. As shown in **Figure 1D**, ANE had no obvious effect on the cell viability of BMMs at the indicated concentration. These findings demonstrate that ANE treatment affect the osteoclast formation without any cytotoxicity.

### ANE Inhibits Bone Resorption Activity and F-Actin Ring Formation

**Figure 2:**
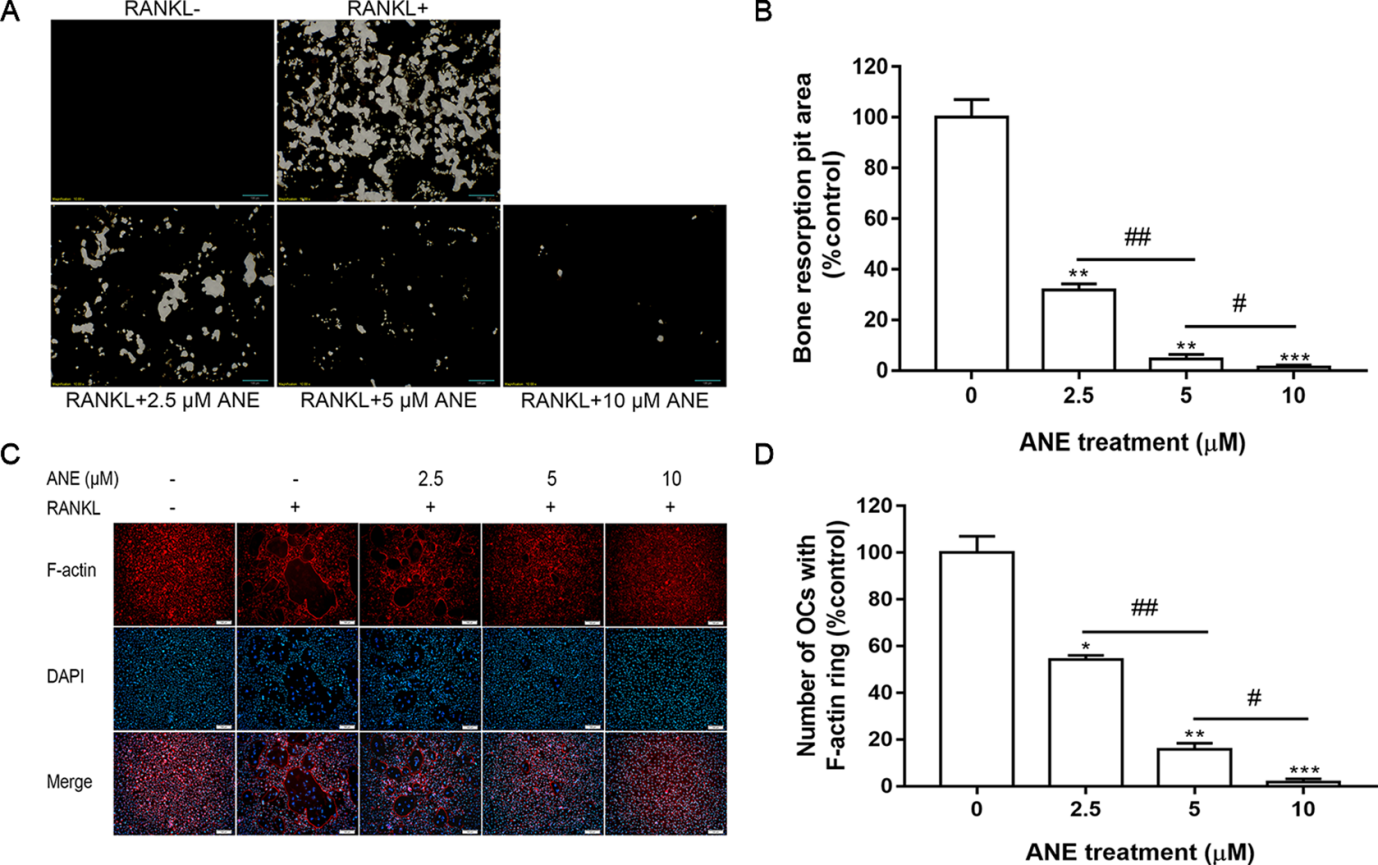
ANE Inhibits Bone Resorption Activity and F-actin Ring Formation. **(A)** BMMs (2 × 105 cells/well) were treated with or without RANKL (100 ng/ml) and ANE (0-10 μM) on 24-well Corning Osteo Assay Surface Plates for 4 days in the presence of M-CSF (40 ng/ml). **(B)** The areas of osteoclast resorption pits were quantified using ImageJ software. **(C)** BMMs (6 × 104 cells/well) were treated with or without RANKL (100 ng/ml) and ANE (0-10 μM) in a 48-well plate for 4 days in the presence of M-CSF (40 ng/ml). Cells were fixed and stained with F-actin rings (Phallodin). **(D)** The number of osteoclasts with F-actin rings was quantified using ImageJ software. Scale bar = 100 μm. Data are presented as mean ± SD (n = 3). Statistical values were calculated using a Student’s t-test unless otherwise indicated. *p < 0.05, **p < 0.01, and ***p < 0.001 vs. RANKL-positive control group. ^#^P < 0.05 and ^##^P < 0.05 vs. the ANE treatment group for the indicated pairwise comparisons. ANE, anemonin; BMMs, bone marrow-derived macrophages; M-CSF, Macrophage Colony Stimulating Factor.

Osteoclasts reorganize the actin cytoskeleton to adhere to mineralized bone surface and to resorb the bone (Garbe et al., 2012). Next, this study examined whether ANE could suppress F-actin ring formation and bone resorption function. As expected, ANE treatment markedly decreased osteoclast-mediated bone resorption pits in a dose-dependent manner (**Figures 2A, B**). Addition, ANE treatment dose-dependently decreased the quantity of F-actin rings and treated cells contained fewer nuclei (**Figures 2C, D**). Taken together, ANE treatment inhibited RANKL-induced osteoclast differentiation and decreased osteoclast size and fusion without obvious cytotoxicity. In addition, it reduced the bone-resorbing function of osteoclasts by impairing F-actin cytoskeleton formation and osteoclastic absorption ability. These results suggest that ANE decreases osteoclast-mediated bone formation and resorption.

### ANE Inhibits RANKL-Induced NFATc1 and c-Fos Expression

**Figure 3:**
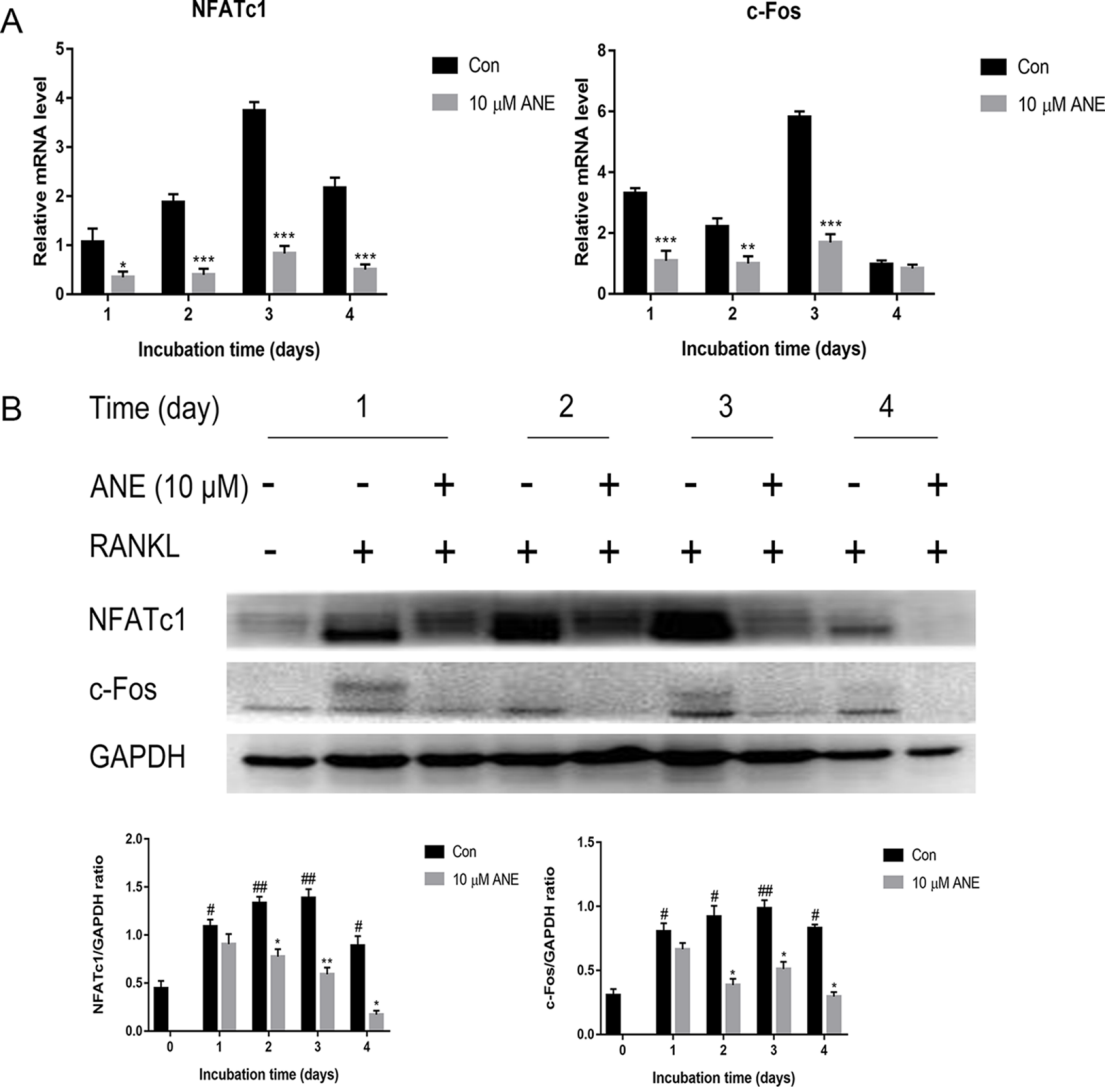
ANE Inhibits RANKL-induced NFATc1 and c-Fos Expression. BMMs were treated with or without 10 μM ANE and RANKL (100 ng/ml) in α-MEM complete medium for 4 days in the presence of M-CSF (40 ng/ml). **(A)** NFATc1 and c-Fos mRNA expression was examined using Real-Time PCR. GAPDH was employed as a reference gene. **(B)** NFATc1 and c-Fos protein expression levels were examined by western blotting using antibodies against NFATc1 and c-Fos. Data are presented as mean ± SD (n = 3). Statistical values were calculated using a Student’s t-test unless otherwise indicated. ^#^p < 0.05 and ^##^p < 0.01 vs. RANKL-negative control group; *p < 0.05, **p < 0.01, and ***p < 0.01 vs. RANKL-positive control group. ANE, anemonin; BMMs, bone marrow-derived macrophages.

Thereafter, this study investigated whether ANE regulates the mRNA and protein expression of NFATc1 and c-Fos. As shown in **Figure 3A**, ANE repressed the mRNA levels of NFATc1 and c-Fos in RANKL-treated BMMs. Similarly, ANE lowered RANKL-stimulated NFATc1 and c-Fos protein level expression (**Figure 3B**). These results suggest the anti-osteoclastogenic effect of ANE may contribute to the inhibition of NFATc1 signaling.

### ANE Downregulates Osteoclast-Specific Gene Expression

**Figure 4:**
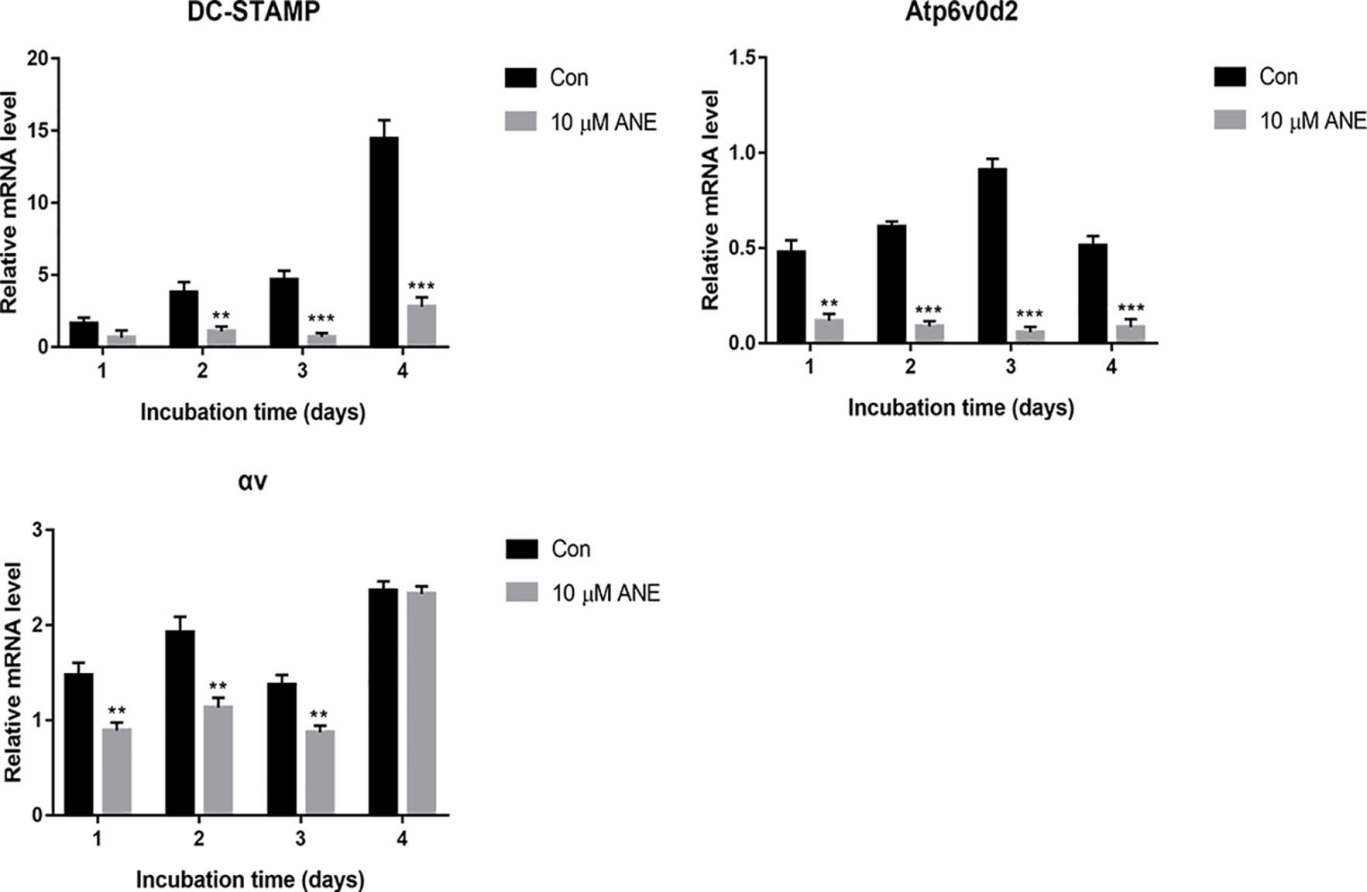
ANE Downregulates Osteoclast-Specific Gene Expression. BMMs were cultured with or without RANKL (100 ng/ml) and 10 μM ANE for the indicated time in the presence of M-CSF (40 ng/ml) and cell samples were collected every day. The mRNA levels of the tested genes were calculated using the comparative Ct (ΔCt) method. Data are presented as mean ± SD (n = 3). Statistical values were calculated using a Student’s t-test unless otherwise indicated. **p < 0.01 and ***p < 0.001 vs. RANKL-positive control group. ANE, anemonin; BMMs, bone marrow-derived macrophages; M-CSF, Macrophage Colony Stimulating Factor.

NFATc1 is a master transcription factor for osteoclastogenesis (Asagiri et al., 2005; Kim and Kim, 2014), which controls the transcription of osteoclastogenesis-essential genes, including ATPase H+-transporting V0 subunit d2 (Atp6v0d2), dendritic cell specific transmembrane protein (DC-STAMP) and Integrin αv. Thus, the study evaluated the expression of these genes by Real-Time PCR. As shown in **Figure 4**, after 24, 48, 72, or 96 h treatment with ANE, the gene expression of DC-STAMP, Integrin αv, and Atp6v0d2 strikingly decreased. These genes are involved in precursor cell fusion and bone resorption (Lee et al., 2006; Chiu and Ritchlin, 2016). Atp6v0d2 and DC-STAMP have been shown to be crucial for cell-to-cell fusion, which is critical for reorganizing the actin cytoskeleton during osteoclast differentiation (Yagi et al., 2005; Kim et al., 2008). Integrin αvβ3 is a transmembrane integrin involved in osteoclast attachment on bone, mediating the downward signaling pathways and subsequent bone resorption (Engleman et al., 1997; Crotti et al., 2006). Taken together, this study provides evidence that ANE might exert its anti-osteoclastogenesis effects *via* inhibition of these NFATc1 responsive genes.

### ANE Attenuates the Activation of the RANKL-Induced NF-κB p65 Phosphorylation

**Figure 5:**
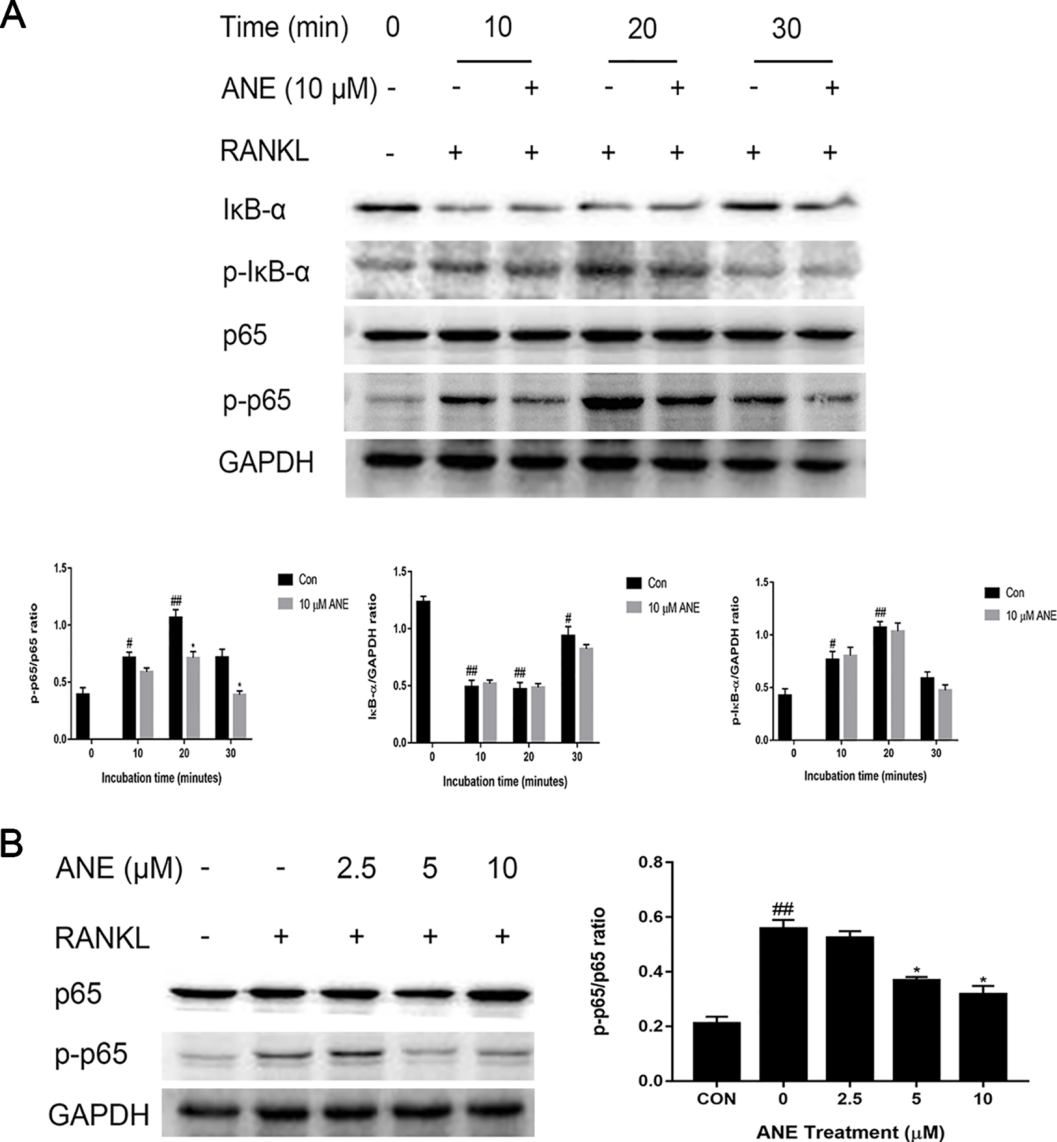
ANE Attenuates the Activation of the RANKL-Induced NF-κB p65 Phosphorylation. **(A)** BMMs were pretreated with 10 μM ANE or DMSO (vehicle) for 1 h and then stimulated with 100 ng/ml RANKL (0–30 min) in the presence of M-CSF (40 ng/ml). Total cell extracts were subjected to western blotting analyses to determine the relative phosphorylation levels of p65 and IκB-α and the degradation of IκB-α. **(B)** BMMs were pretreated with ANE (0-10 μM) or DMSO (vehicle) and then stimulated with 100 ng/ml RANKL in the presence of M-CSF (40 ng/ml) for 20 min. The relative expression levels of the target proteins were quantified using ImageJ software. GAPDH is designed as loading control. Data are presented as mean ± SD (n = 3). Statistical values were calculated using a Student’s t-test unless otherwise indicated. ^#^p < 0.05 and ^##^p < 0.01 vs. RANKL-negative control group; *p < 0.05 vs. RANKL-positive control group. ANE, anemonin; BMMs, bone marrow-derived macrophages; DMSO, dimethyl sulfoxide; M-CSF, Macrophage Colony Stimulating Factor.

As noted in many studies, NF-κB signaling activation is important for the initial induction of NFATc1, ultimately resulting in RANKL-induced osteoclast differentiation (Ono and Nakashima, 2018). To investigate whether ANE affected NFATc1 induction through NF-κB signaling, this study next examined the influence of ANE on RANKL-induced NF-κB activation by western blotting analysis. BMMs were pretreated with different concentrations of ANE for 1 h and then incubated with RANKL for the indicated time. RANKL-mediated p65 phosphorylation was considerably decreased by ANE treatment at 20 min of RANKL stimulation, while IκB-α degradation and phosphorylation was relatively unaffected in the ANE group (**Figure 5A**). Additionally, ANE pretreatment strongly reduced p65 phosphorylation in a concentration-dependent manner at 20 min RANKL stimulation (**Figure 5B**). These findings demonstrate that ANE treatment reduced RANKL-induced NF-κB activation through IκB-α independent manner.

### ANE Inhibits RANKL-Induced ERK1/2 Signaling

**Figure 6:**
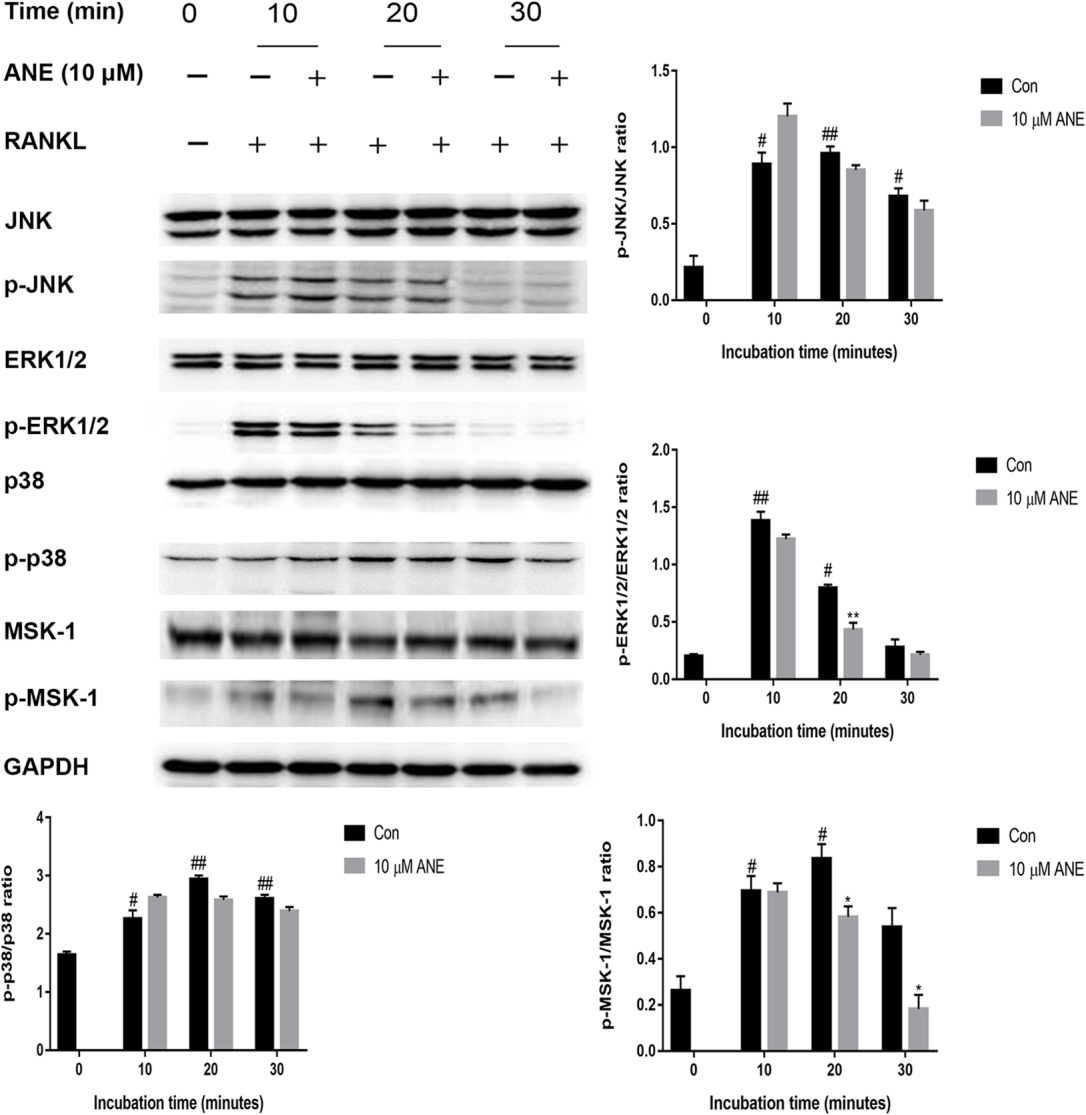
ANE inhibits RANKL-induced phosphorylation levels of ERK1/2 and MSK-1. BMMs were pretreated with 10 μM ANE or DMSO (vehicle) for 1 h and then stimulated with 100 ng/ml RANKL (0–30 min) in the presence of M-CSF (40 ng/ml). Total cell extracts were subjected to western blotting analyses to determine the relative phosphorylation levels of ERK1/2, p38, JNK, and MSK-1. GAPDH is designed as loading control. The relative expression levels of the target proteins were quantified using ImageJ software. Data are presented as mean ± SD (n = 3). Statistical values were calculated using a Student’s t-test unless otherwise indicated. ^#^p < 0.05 and ^##^p < 0.01 vs. RANKL-negative control group; *p < 0.05 and **p < 0.01 vs. RANKL-positive control group. ANE, anemonin; BMMs, bone marrow-derived macrophages; DMSO, dimethyl sulfoxide; M-CSF, Macrophage Colony Stimulating Factor.

MAPK signaling which consists of three major members p38, ERK1/2, and JNK, plays a crucial role in osteoclast differentiation, have shown to be activated in RANKL-induced osteoclastogenesis (Nakashima et al., 2012; Choi et al., 2017). To investigate whether ANE affects the MAPK pathways, BMMs pretreated with the indicated concentration of ANE for 1 h and then stimulated with RANKL for different times (0 to 30 min). The expression of phosphorylated ERK1/2 was significantly reduced compared with total ERK1/2 by ANE treatment at 20 min of RANKL stimulation. However, the relative expression of phosphorylated p38 and JNK was relatively unaffected in the ANE group (**Figure 6**). Previous studies also reported that MSK1, a downstream kinase of ERK1/2, positively regulates p65 signaling pathways (Schmitz et al., 2001; Vermeulen et al., 2003). To investigate whether MSK1 is involved in mediating the phosphorylation of p65 in the ANE-treated osteoclasts, this study checked the phosphorylation of MSK-1. As shown in **Figure 6**, the phosphorylation of MSK-1 was significantly reduced compared with total MSK-1 at 20 min of ANE treatment. These data suggest that ANE inhibits RANKL-mediated activation of the ERK1/2 signaling pathway without changing JNK and p38 signaling pathways.

### ANE Does Not Affect RANKL-Induced Phosphorylation of PLCγ2 and GSK-3β

**Figure 7:**
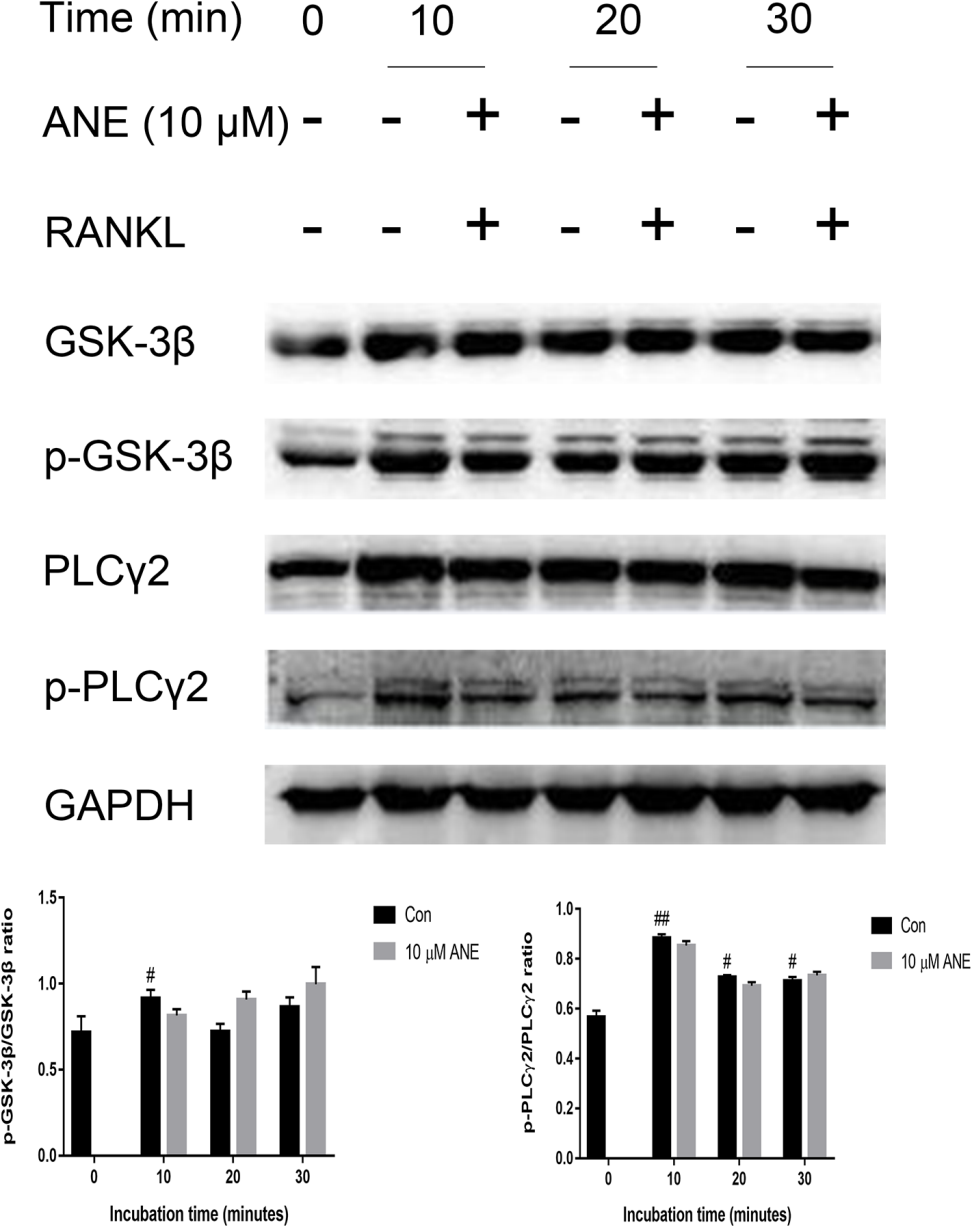
ANE does not affect the phosphorylation levels of PLCγ2 and GSK-3β. **(A)** BMMs were pretreated with 10 μM ANE or DMSO (vehicle) for 1 h and then stimulated with 100 ng/ml RANKL (0–30 min) in the presence of M-CSF (40 ng/ml). Total cell extracts were prepared for analysis of the relative phosphorylation levels of PLCγ2 and GSK-3β. The GAPDH expression is used as loading control. The relative expression levels of the target proteins were quantified using ImageJ software. Data are presented as mean ± SD (n = 3). Statistical values were calculated using a Student’s t-test unless otherwise indicated. ^#^p < 0.05 and ^##^p < 0.01 vs. RANKL-negative control group. ANE, anemonin; BMMs, bone marrow-derived macrophages; DMSO, dimethyl sulfoxide; M-CSF, Macrophage Colony Stimulating Factor.

In addition, the study provides insights into the effect of ANE on the regulation of NFATc1 in osteoclasts. To investigate whether ANE can modulate NFTAc1 phosphorylation, this study examined the effect of ANE on the activation of GSK-3β and PLCγ2. As shown in **Figure 7**, RANKL induced inactivation of GSK-3β and activation PLCγ2, which were not affected by ANE treatment. Taken together, these results suggest that ANE treatment might not affect the phosphorylation modification of NFATc1 mediated by GSK-3β and PLCγ2 signaling.

### ANE Regulates the Expression of Transcriptional Repressors Blimp1, Bcl-6, and IRF-8

**Figure 8:**
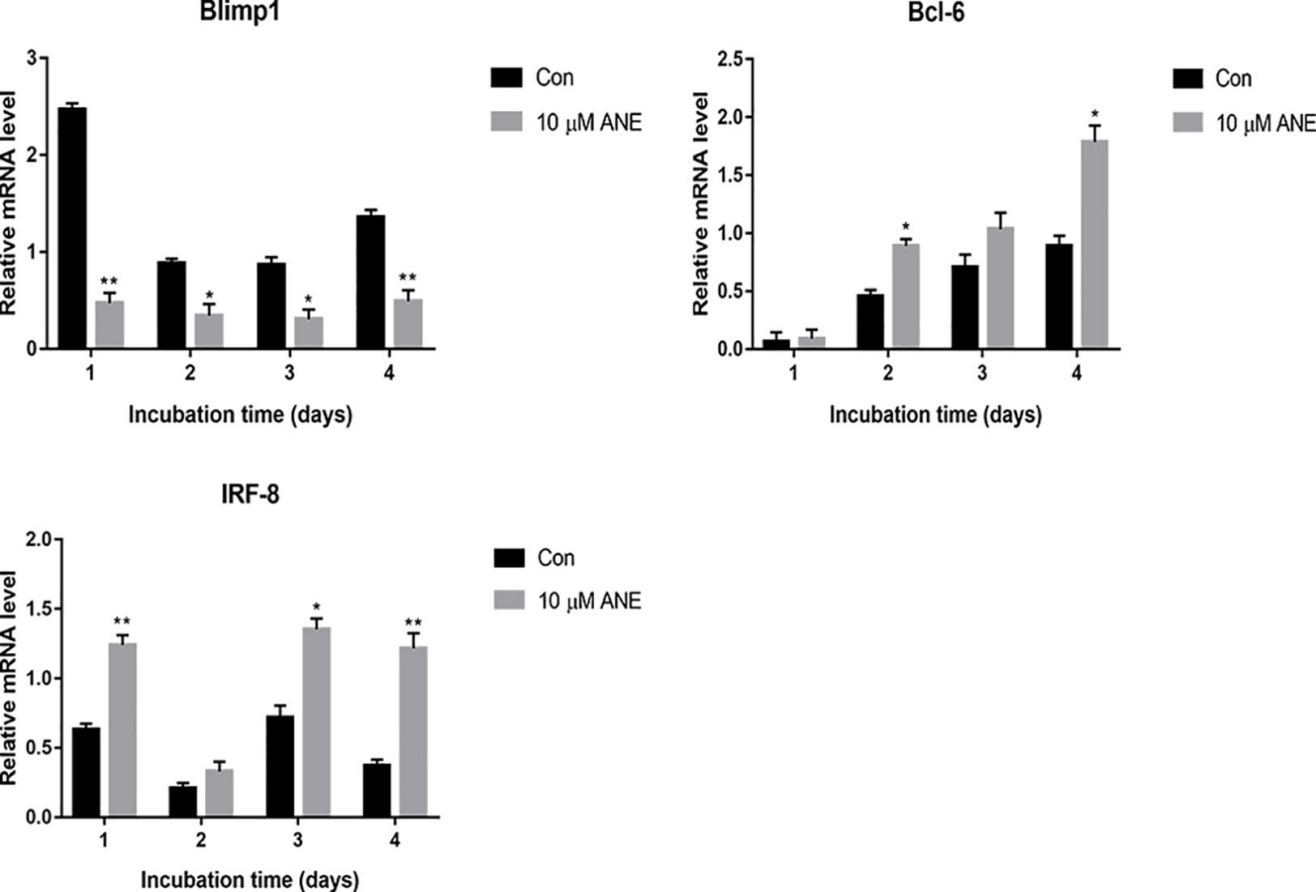
ANE regulates the mRNA expression of transcriptional repressors Blimp1, Bcl-6, and IRF-8. BMMs were cultured with or without RANKL (100 ng/ml) and 10 μM ANE for the indicated time in the presence of M-CSF (40 ng/ml) and cell samples were collected every day. The mRNA levels of Blimp1, Bcl-6, and IRF-8 were determined by Real-Time PCR and calculated using the comparative Ct (ΔCt) method. Data are presented as mean ± SD (n = 3). Statistical values were calculated using a Student’s t-test unless otherwise indicated. *p < 0.05 and **p < 0.01 vs. RANKL-positive control group. ANE, anemonin; BMMs, bone marrow-derived macrophages; M-CSF, Macrophage Colony Stimulating Factor.

Previous studies have reported that Blimp1 (Prdm1), a transcriptional repressor, is necessary for osteoclastogenesis (Nishikawa et al., 2010). NFATc1 expression is downregulated by transcriptional repressors such as IRF-8 and Bcl-6; Blimp-1 negatively regulates IRF-8 and Bcl-6 (Zhao and Ivashkiv, 2011). Thus, this study further examined whether ANE could affect the expression of these regulators of NFATc1. As shown in **Figure 8**, ANE treatment markedly decreased the expression of Blimp1, while increasing the expression of IRF-8, and Bcl-6. These results indicate that ANE might suppress NFTAc1 transcription *via* inhibition of Blimp1 followed by enhancement of Bcl-6 and IRF-8. Taken together, these findings indicate that ANE might inhibit NFATc1 activation by modulating NF-κB and ERK1/2 signaling and enhancing the expression of NFATc1 negative regulators Bcl-6 and IRF-8.

### ANE Inhibits LPS-Induced Bone Loss in Mice

**Figure 9:**
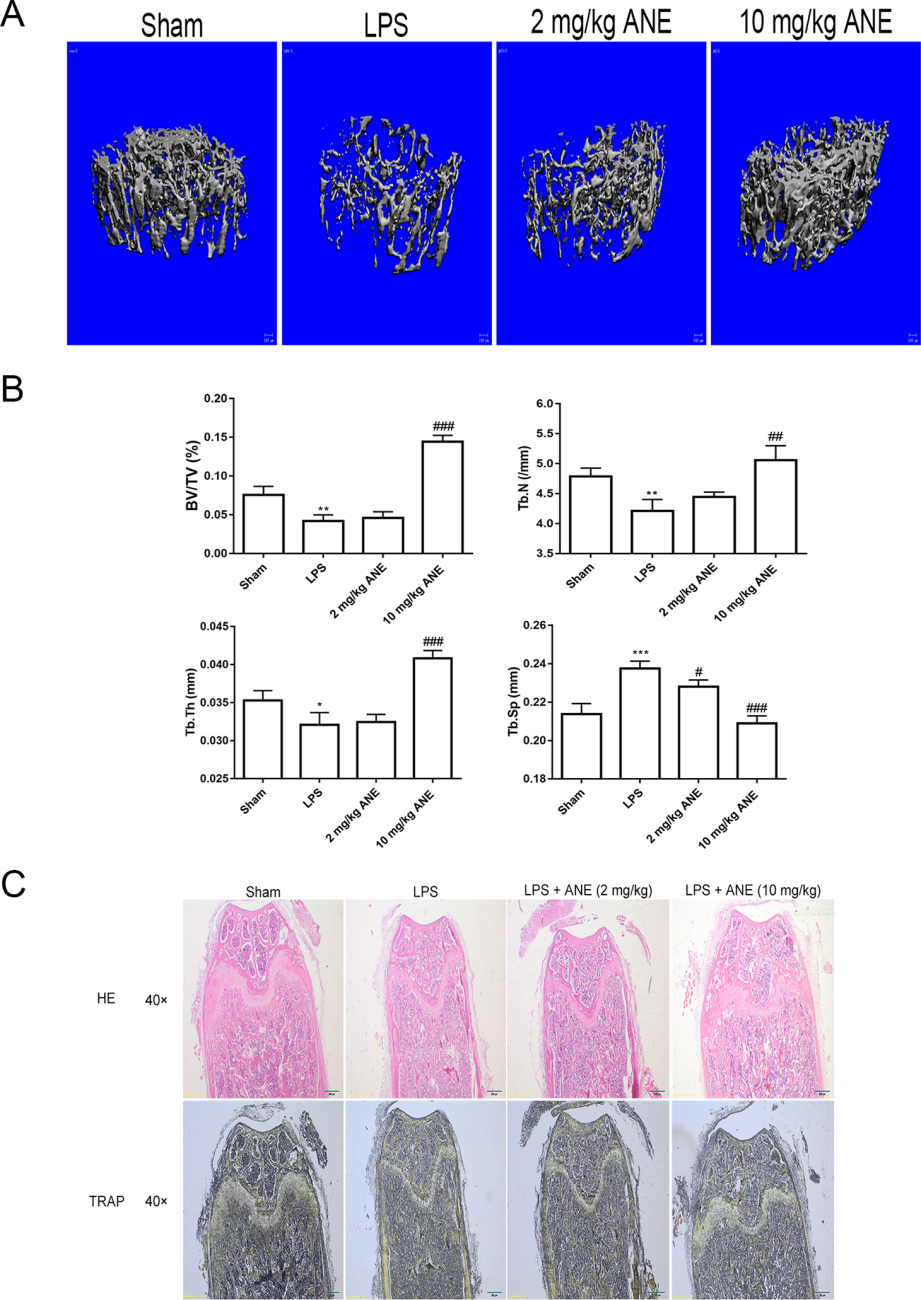
ANE Inhibits LPS-Induced Bone Loss in Mice. **(A)** Three-dimensional reconstructed images of the right femora from each treatment group. **(B)** Micro-CT analyses of the bone parameters BV/TV, Tb.N, Tb.Th, and Tb.Sp. All bar graphs are presented as mean ± SD (n = 10). **(C)** Representative images of femur sections stained with H&E and TRAP. *p < 0.05, **p < 0.01, and ***p < 0.001 vs. SHAM group; ^#^p < 0.05, ^##^p < 0.01, and ^###^p < 0.001 vs. LPS group. Statistical values were calculated by ANOVA followed by *post hoc* Bonferroni test. ANE, anemonin; BV/TV, bone volume per tissue volume; Tb.Sp, trabecular separation; Tb.Th, trabecular thickness; Tb.N., trabecular number.

Owing to these promising cellular effects, the effects of ANE on LPS-treated bone loss in a mouse model were evaluated using micro-CT and histology. Compared with the LPS group, bone microfracture parameters, including trabecular BV/TV, Tb.N, Tb.Th, and Tb.Sp, were significantly improved in the ANE high-dose group (**Figures 9A, B**), while the ANE low-dose groups did not shown an obvious effect. H&E and TRAP staining showed that ANE treatment obviously reduced LPS-induced bone fracture destruction (**Figure 9C**). Briefly, morphometric and histomorphometric analyses showed that ANE treatment significantly improved BV/TV and trabecular bone parameters and improved bone destruction in LPS-induced mice. These results indicate that ANE may have the potential to protect against bone loss.

## Discussion

Osteoclast differentiation is a complex mechanism that comprises cell fusion and maturation triggered after RANKL binds to RANK (Boyle et al., 2003; Asagiri and Takayanagi, 2007). This study demonstrated that ANE can inhibit osteoclast differentiation and activity *via* regulation of RANKL-induced NFATc1 expression. Furthermore, *in vivo* studies indicated that ANE can improve LPS-mediated inflammatory bone loss in mice.

ANE treatment inhibited RANKL-induced osteoclast differentiation, decreased osteoclast size and fusion without obvious cytotoxicity. In addition, it reduced the bone-resorbing function of osteoclasts through impairing the F-actin cytoskeleton formation and osteoclastic absorption ability. NFATc1 and c-Fos are crucial transcription factors for osteoclastogenesis (Asagiri et al., 2005; Kim and Kim, 2014). In this study, ANE treatment markedly blocked c-Fos and NFATc1 transcriptional activities and protein expression caused by RANKL. Consistently, it also decreased the expression of NFATc1 responsive genes, including Atp6v0d2, DC-STAMP and αv. Atp6v0d2 and DC-STAMP identified as crucial for cell fusion (Yagi et al., 2005; Kim et al., 2008). Integrin αvβ3 is a transmembrane integrin which involved in osteoclast attachment on bone, mediating the downward signaling pathways and subsequent bone resorption (Engleman et al., 1997; Crotti et al., 2006). Together, these data supplied evidence for the anti-osteoclastogenic effect of ANE may be in part *via* the inhibition of NFATc1 signaling.

As noted in many studies, activation of MAPKs and NF-κB signaling is important for the initial induction of NFATc1, ultimately resulting in RANKL-induced osteoclast differentiation (Ono and Nakashima, 2018). Previous studies indicated ERK1/2 phosphorylation plays important roles in osteoclast precursor cells proliferation, survival and osteoclast differentiation (Nakamura et al., 2003; Lee et al., 2009). The activation of ERK1/2 could promote the expression of transcription factor c-Fos, which is component of transcription factor complex AP-1 and enhanced the expression of master transcription factor NFATc1 (Miyazaki et al., 2000; Nakamura et al., 2003; Zupan et al., 2013). Pharmacological inhibition of ERK1/2 activity with selective inhibitor PD98059 markedly blocked osteoclast formation without affecting bone resorption function of mature osteoclasts (Nakamura et al., 2003; Kim et al., 2014). These results indicated that ANE might reduce NFATc1 expression through attenuating activation of ERK1/2 and its downstreaming target gene c-Fos. For canonical NF-κB signaling pathway, in rest or unstimulated conditions, NF-κB p50-p65 heterodimer is bound to its inhibitor IκB-α. The activation of NF-κB requires the release of NF-κB from inhibitors of NF-κB (IκBs) in cytoplasma. Upon external stimuli, such as TNF-α, LPS, and RANKL, IκB-α is phosphorylated and degraded, NF-κB dimer is released and translocated to the nucleus, where it promotes the transcription of its target genes *via* binding to promoter of these genes. Current study demonstrates that ANE treatment reduced RANKL-induced NF-κB activation through IκB-α independent manner. Besides IκB-dependent manner, the posttranslational modification of NF-κB DNA binding subunits such as p65, also directly regulates the transcription factor function of NF-κB. Several signaling pathways have a crosstalk with NF-κB signaling through directly or indirectly regulating phosphorylation of p65. Previous studies were demonstrated that the phosphorylation of p65 is a prerequisite for the transcriptional activity of NF-κB (Zhong et al., 1998; Bohuslav et al., 2004; Sasaki et al., 2005; Lu and Yarbrough, 2015). In this study, the phosphorylation of p65 was examined. More importantly, ANE treatment can partly suppressed p65 phosphorylation without altering the phosphorylation and degradation of IκB-α. It was reported that phosphorylation of p65 subunit can be phosphorylated by mitogen and stress-activated kinase 1 (MSK-1), which is the downstream target of ERK1/2 and p38 MAPK (Schmitz et al., 2001; Vermeulen et al., 2003). The inhibitors of ERK1/2 or p38 ablated serine residues of p65 through suppression of MSK-1 phosphorylation (Kefaloyianni et al., 2006). Consistent with this observation, these results also showed that ANE could reduce ERK1/2 phosphorylation and MSK-1 phosphorylation. So it can be suggested that ANE could affect the phosphorylation of p65 *via* ERK1/2-MSK-1 signaling pathway. Taking together, these data implicated that ANE might modulate NF-κB p65 phosphorylation in IκB-α-independent manner.

Besides the initial induction of NFATc1 by NF-κB and MAPK signaling at the early stages of osteoclastogenesis, the modulatory phosphorylation of NFATc1 also directly affects the transcriptional activity (Asagiri et al., 2005). NFTAc1 phosphorylation is known to lead to inactivation of this master transcription factor of osteoclastogenesis. GSK-3β can directly phosphorylate NFATc1, which is needed for nuclear export, and promotes nuclear exit (Jang et al., 2011). Calcineurin enhances NFATc1 activity by dephosphorylating NFATc1. The activation of PLCγ2-Ca2+ signaling increases the expression of calcineurin (Takayanagi et al., 2002; Asagiri and Takayanagi, 2007). These results showed that ANE did not change activation of GSK-3β and PLCγ2, which implicated that ANE might not affect the phosphorylation modification of NFATc1.

Previous studies have reported that several factors, including Bcl-6 and IRF-8, negatively regulate NFTAc1 transcription. IRF-8 and Bcl-6 inhibits auto-amplification of NFATc1 and its transcriptional activity (Zhao and Ivashkiv, 2011). Another protein, Blimp-1, a transcriptional repressor of the negative regulators of NFATc1, has also been reported to reduce the expression of Bcl-6 and IRF-8 by suppressing their transcription, which is essential for osteoclastogenesis (Nishikawa et al., 2010; Zhao and Ivashkiv, 2011). In this study, the data from Real-Time PCR shows that ANE downregulated RANKL-induced Blimp-1 expression, while limiting the reduction of the negative regulators IRF-8 and Bcl-6. However, the exact underlying mechanism deserves further investigation in the future. Taken together, these findings indicate that ANE might inhibit NFATc1 activation by modulating NF-κB and ERK1/2 signaling and enhancing the expression of NFATc1 negative regulators Bcl-6 and IRF-8.

Owing to these promising cellular effects, this study next explored the latent therapeutic effect of ANE on LPS-induced osteoporosis *in vivo*. Morphometric and histomorphometric analyses showed that ANE treatment significantly improved BV/TV and trabecular bone parameters and improved bone destruction in LPS-induced mice. These results indicate that ANE may have the potential to protect against bone loss.

In summary, this study demonstrates that ANE treatment has anti-osteoclastogenic and anti-bone resorptive effects *in vitro* and *in vivo*. Furthermore, ANE has the potential to protect against inflammatory bone loss in mice. Taken together, ANE could serve as a novel anti-resorptive agent against osteoclast-related diseases.

## Data Availability Statement

The raw data supporting the conclusions of this article will be made available by the authors, without undue reservation, to any qualified researcher.

## Ethics Statement

The animal study was reviewed and approved by the Institutional Animal Ethics Committee of Jilin University.

## Author Contributions

YW, W-CS, and H-BW conceived and designed the experiments. HH, QP, SW, YD, JC, and YZ performed the experiments and analyzed the data. HH and W-CS wrote the manuscript. All authors read and approved the final manuscript.

## Funding

This work was supported by the National Natural Science Foundation (31670347, 81001369, 31170327, and 31870121), Jilin Province Science and Technology Department (20170311015YY, 20190201003JC), and Jilin Provincial Health Special Project (2019SCZ063).

## Conflict of Interest

The authors declare that the research was conducted in the absence of any commercial or financial relationships that could be construed as a potential conflict of interest.
